# SRSF5‐Mediated Alternative Splicing of M Gene is Essential for Influenza A Virus Replication: A Host‐Directed Target Against Influenza Virus

**DOI:** 10.1002/advs.202203088

**Published:** 2022-10-18

**Authors:** Qiuchen Li, Zhimin Jiang, Shuning Ren, Hui Guo, Zhimin Song, Saini Chen, Xintao Gao, Fanfeng Meng, Junda Zhu, Litao Liu, Qi Tong, Honglei Sun, Yipeng Sun, Juan Pu, Kin‐Chow Chang, Jinhua Liu

**Affiliations:** ^1^ Key Laboratory for Prevention and Control of Avian Influenza and Other Major Poultry Diseases Key Laboratory of Animal Epidemiology Ministry of Agriculture College of Veterinary Medicine China Agricultural University Beijing 100193 China; ^2^ Chinese Academy of Sciences Key Laboratory of Infection and Immunity Institute of Biophysics Chinese Academy of Sciences Beijing 100101 China; ^3^ Biotechnology Research Institute Chinese Academy of Agricultural Sciences Beijing 100081 China; ^4^ School of Veterinary Medicine and Science University of Nottingham Sutton Bonington Campus Sutton Bonington LE12 5RD UK

**Keywords:** alternative splicing, anidulafungin, influenza A viruses, SRSF5, U1 snRNP

## Abstract

Splicing of influenza A virus (IAV) RNA is an essential process in the viral life cycle that involves the co‐opting of host factors. Here, it is demonstrated that induction of host serine and arginine‐rich splicing factor 5 (SRSF5) by IAV facilitated viral replication by enhancing viral M mRNA splicing. Mechanistically, SRSF5 with its RRM2 domain directly bounds M mRNA at conserved sites (M mRNA position 163, 709, and 712), and interacts with U1 small nuclear ribonucleoprotein (snRNP) to promote M mRNA splicing and M2 production. Mutations introduced to the three binding sites, without changing amino acid code, significantly attenuates virus replication and pathogenesis in vivo. Likewise, *SRSF5* conditional knockout in the lung protects mice against lethal IAV challenge. Furthermore, anidulafungin, an approved antifungal drug, is identified as an inhibitor of SRSF5 that effectively blocks IAV replication in vitro and in vivo. In conclusion, SRSF5 as an activator of M mRNA splicing promotes IAV replication and is a host‐derived antiviral target.

## Graphical abstract

Influenza A viruses (IAVs) must depend on the host splicing machinery to syntheses various essential proteins. The study uncovers the important role of host protein SRSF5 in activating the splicing of influenza viral M messenger RNA. Using a virtual structure‐based drug screening, anidulafungin is identified to target SRSF5 and shows potential for the inhibition of IAVs infections.

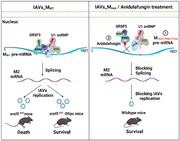

## 1 Introduction

Influenza A viruses (IAVs) are prevalent in many mammalian and avian host species, and periodically cause epidemics or pandemics in humans, and epizootics or panzootics in animals.^[^ ^1^ ^]^ Globally each year, there are around one billion human infections of which 3–5 million are clinically critical with 300 000–600 000 ensuing deaths (https://www.cdc.gov/flu/season/index.html). Presently, with the ongoing COVID‐19 pandemic, there are concerns of reduced population immunity against influenza viruses from prolonged shielding and reduced exposure. Annual influenza vaccination is still the most effective protection available against the highly mutable virus. All antivirals that target specific influenza viral components (such as M2 ion channel, neuraminidase, and polymerase subunit PA) are known to readily cause virus resistance which greatly limits their usefulness.^[^ ^2^ ^]^ Rather than directly targeting viral components, it has been suggested that a cell‐based approach that targets key host‐dependent factor(s) necessary for viral replication could confer reduced virus resistance and broad‐spectrum efficacy.^[^ ^3^ ^]^ Recently, it was found that thapsigargin, a sarco‐endoplasmic Ca^2+^‐ATPase pump inhibitor, is a highly potent broad‐spectrum host‐centric antiviral against three major human respiratory viruses: influenza A virus, SARS‐CoV‐2, and respiratory syncytial virus.^[^ ^4^ ^]^

In eukaryotic cells, pre‐mRNA alternative splicing (AS) is commonly used to produce different transcripts (and therefore proteins) from a single gene. Some viruses that replicate in the host nucleus have usurped host AS mechanisms to expand the coding capacity of their limited gene segments to increase viral protein types.^[^ ^5^ ^]^ In IAV, NS, and M genes, the two smallest segments in the viral genome, are alternatively spliced at nuclear speckle sites that result in the added generation of nonstructural protein 2 (NS2) and M2 ion channel protein respectively.^[^ ^6^ ^]^ The M gene can additionally be spliced to produce M3 and M4 mRNA; the latter encodes M42 a novel ion channel protein.^[^ ^7^ ^]^ Viral proteins derived from AS are known to perform essential roles in viral replication and infection, such as regulating nuclear export of IAV mRNAs, viral budding and adaptation.^[^ ^8^ ^]^ IAV has evolved specific regulatory mechanisms of host splicing machinery to enable the expression of specific viral products during virus infection.^[^ ^9^ ^]^ Thus, exogenous modulation of splicing to target specific key regulator(s) could be an effective host‐based antiviral approach. However, key host regulators involved in IAV mRNA AS have not been fully elucidated.

Each protein member of the serine and arginine rich (SR) protein family, comprising 12 classical SR‐splicing factors (SRSF1 to SRSF12) and RNA binding SR‐like splicing factors,^[^ ^10^ ^]^ contains an arginine‐serine‐rich (RS) protein binding domain, and one or two RNA recognition motif(s) (RRM).^[^ ^11^ ^]^ SRSFs are mainly located in nuclear speckles and function in constitutive splicing and alternative splicing of cellular genes;^[^ ^6^, ^12^ ^]^ they are increasingly shown to be intimately involved in viral RNA splicing during infection. In human adenovirus IIIa gene, AS is repressed by SRSF1, SRSF2, and SRSF6 through their binding to an intronic repressor element and impeding the recruitment of U2 small nuclear ribonucleoprotein (snRNP) to the spliceosome.^[^ ^13^ ^]^ In human papillomavirus type 16 infection, SRSF3 is induced to support viral L1 mRNA splicing and capsid protein expression.^[^ ^14^ ^]^ Herpes simplex virus protein ICP27 has been shown to inactivate SR protein kinase 1, leading to hypophosphorylation inhibition of SRSF1‐mediated splicing.^[^ ^15^ ^]^ Expression of four essential human immunodeficiency virus‐1 (HIV‐1) structural and regulatory proteins (Gag, Env, Tat, and Rev) during infection are blocked by the compound 5 342 191 that targets SRSF1, 3, and 4.^[^ ^16^ ^]^ We previously found that SR proteins (SRSF1, SRSF2, SRSF3, SRSF5, SRSF6, and SRSF7) are able to bind to influenza viral RNA using an affinity proteomics approach with viral RNA as bait.^[^ ^17^ ^]^ However, the precise role of SR proteins in IAV RNA splicing remains unclear.

Here, we examined the functionality SR proteins in influenza virus infection and established that SRSF5 promotes virus replication in vitro and in vivo via boosting viral M mRNA AS to improve M2 production. We found that SRSF5, via its RRM2 domain, directly binds M mRNA and identified two critical SRSF5‐binding motifs in the M pre‐mRNA. We further demonstrated that during IAV infection SRSF5 recruits, via U1A binding, U1 snRNP to the M pre‐mRNA. Finally, from virtual structure‐based drug screening, we showed that anidulafungin, an approved antifungal agent, targets the SRSF5 RRM2 domain that results in IAV inhibition in vitro and in vivo.

## 2 Results

### 2.1 IAV Infection Increased SRSF5 mRNA and Protein Expression

**Figure 1:**
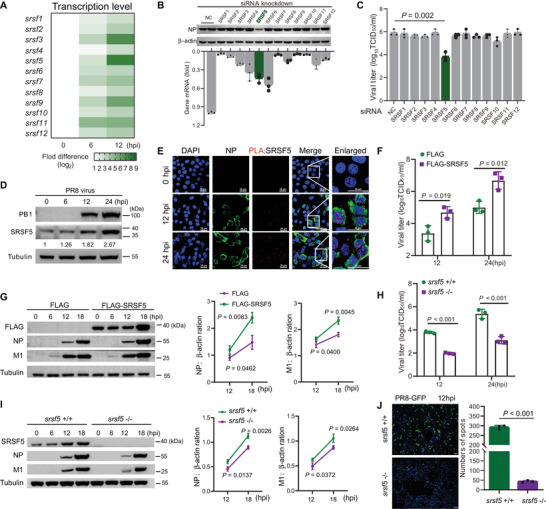
SRSF5 was highly up‐regulated during IAV infection and involved in IAV replication. A) mRNA levels, detected by RT‐qPCR, of 12 *SRSF*s in A549 cells infected with PR8 virus at 1.0 MOI were determined at 0, 6, and 12 hpi. B) A549 cells were transfected with specific SR protein‐targeting siRNAs or negative control (NC) siRNA for 24 h, followed by infection with PR8 virus (MOI = 1). mRNA expression of *SRSF*s was determined by RT‐qPCR (bottom). Viral NP protein expression was determined by Western blotting at 18 hpi. *β*‐Actin detection was used as a loading control (top). C) A549 cells were transfected with SR protein‐targeting siRNAs or negative control (NC) siRNA for 24 h followed by infection with PR8 virus (MOI = 1). Viral titers were measured by TCID_50_ assay at 18 hpi. Data presented as means ± SD and are representative of three independent experiments. D) SRSF5 protein expression (fold‐change indicated) in PR8 virus‐infected (1.0 MOI) A549 cells at 0, 6, 12, and 24 hpi. Tubulin was used as a loading control. E) Intracellular localization of SRSF5 in PR8 virus‐infected A549 cells at 0, 12, and 24 hpi by in situ PLA assay. Scale bars, 255 µm. F) A549 cells were transfected with SRSF5‐Flag plasmids or empty vector control for 24 h and infected with PR8 virus at 1.0 MOI. Viral titers were determined by TCID_50_ assays at the indicated time points. Data presented as means ± SD and are representative of three independent experiments. G) A549 cells were transfected with SRSF5‐Flag plasmids or empty vector control for 24 h and infected with PR8 virus at 1.0 MOI. NP and M1 proteins were detected by Western blotting at 0, 6, 12, and 18 hpi; tubulin detection was used as loading control. NP and M1 proteins are presented as ratios to tubulin; data represent means ± SD (*n* =  3 independent experiments). H) Viral titers in PR8 virus‐infected (1.0 MOI) *srsf5* ^+/+^ and *srsf5* ^−/−^ HEK293 cells were determined by TCID_50_ assays at the indicated time points. Data presented as means ± SD and are representative of three independent experiments. I) NP and M1 proteins from PR8 virus‐infected *srsf5* ^+/+^ and *srsf5* ^−/−^ HEK293 cells were detected by Western blotting at 0, 6, 12, and 18 hpi; tubulin detection was used as loading control. NP and M1 proteins are presented as ratios to tubulin; data represent means ± SD (*n* = 3 independent experiments). J) Fluorescence detection of viral replication (green) in *srsf5* ^+/+^ and *srsf5* ^−/−^ HEK293 cells after infection with GFP‐PR8 virus for 12 h. Scale bars, 100 µm.

In previous research, we performed affinity purification coupled with mass spectrometry (AP‐MS) to uncover the interactions between influenza A virus RNA and host protein using transcribed and biotin‐labeled virus RNA.^[^ ^17^ ^]^ Interestingly, domain‐enrichment analysis showed spliceosome was one of the most associated binding components among the isolated RBPs (Figure S1A, Supporting Information). Several of splicing factor proteins were identified, including *SRSF1*, *SRSF2*, *SRSF3*, *SRSF5*, *SRSF6*, and *SRSF7*.^[^ ^17^ ^]^ In order to investigate whether *SRSF*s proteins were involved in IAV infection, we infected A549 human lung epithelial cells with PR8 virus at 1.0 MOI to determine levels of mRNA of 12 *SRSF*s by qRT‐PCR at 0, 6, and 12 h postinfection (hpi). mRNAs of all *SRSF*s were up‐regulated at 6 and 12 hpi. Notably, *SRSF3* and *SRSF5* mRNAs displayed the highest increase in expression (**Figure** **1**A). Subsequently, separate siRNA knockdown of *SRSF*s in A549 cells, followed by PR8 virus infection, showed that only *SRSF5* knockdown resulted in significant reduction in viral *NP* protein and progeny virus production which suggests that SRSF5 facilitates PR8 virus replication (Figure 1B,C). Therefore, we focused on the SRSF5 protein and western blotting further showed rising SRSF5 protein levels in an infection time‐course dependent manner of 6, 12, and 24 hpi (Figure 1D). In proximity ligation assays (PLAs), abundance of SRSF5 positive spots were clearly elevated in the nuclei of A549 cells at 12 and 24 hpi (Figure 1E). To examine *SRSF5* expression during infection in vivo, mice were separately infected with PR8, H9N2 and H5N1 viruses, each mouse given a median tissue culture infectious dose (TCID_50_) of 10^3^ in 50 µL. Each virus at 3 day postinfection (dpi) induced significant increase in *SRSF5* expression (by ≈3‐fold) in infected lungs (Figure S1B, Supporting Information). Together, these results demonstrate that the *SRSF5* mRNA and protein are induced by IAV infection.

### 2.2 SRSF5 Promoted IAV Replication In Vitro and In Vivo

To determine the impact of SRSF5 on influenza virus replication, SRSF5‐Flag expression plasmid was transfected into A549 cells followed by PR8 virus infection at 1.0 MOI. Over‐expression of SRSF5 significantly increased viral titers at 12 and 24 hpi (Figure 1F) and elevated viral NP and M1 protein expression (Figure 1G). Conversely, *SRSF5* knockdown significantly reduced viral protein detection (Figure S1C and S1D, Supporting Information). Next, *srsf5* ^+/+^ and *srsf5* ^−/−^ HEK293 cells were infected with PR8 virus at 1.0 MOI. Infected *srsf5* ^−/−^ HEK293 cells produced significantly less progeny virus at 12 and 24 hpi (Figure 1H) and less viral NP and M1 protein at 12 and 18 hpi (Figure 1I). Additionally, replication of green fluorescent protein (GFP)‐tagged PR8 virus was markedly reduced, based on fluorescence, in *srsf5* ^−/−^ HEK293 cells relative to *srsf5* ^+/+^ HEK293 cells (Figure 1J). Additional virus subtypes, avian H9N2 and H5N1 virus, were used to infect *srsf5* ^+/+^ and *srsf5* ^−/−^ HEK293 cells at 1.0 MOI. Infected *srsf5* ^−/−^ HEK293 cells had reduced relative expression of H9N2 NP, M1, and PB1 proteins at 6, 12, and 24 hpi (Figure S1E, Supporting Information) and had significantly reduced viral titers of H9N2 and H5N1 at 18 hpi (Figure S1F and S1G, Supporting Information). Collectively, SRSF5 promotes influenza virus replication in human cells.

**Figure 2:**
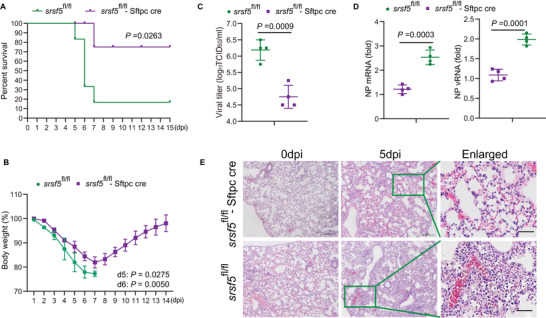
SRSF5‐deficient mice are resistant to influenza virus infection. A) Survival of *srsf5* ^fl/fl^‐Sftpc cre and *srsf5* ^fl/fl^ mice infected with 1×10^2^ TCID_50_ of PR8 virus (*n* = 6). Data presented were pooled from three independent experiments. Kaplan–Meier survival curves subjected to log‐rank (Mantel–Cox) analysis. B) *srsf5* ^fl/fl^‐Sftpc cre and *srsf5* ^fl/fl^ mice (*n*  =  4) were infected with 100 TCID_50_ of PR8 virus. Body weight was monitored daily. Data are presented as the mean ± SD. C) Viral titers in lung tissues from PR8‐virus‐infected *srsf5* ^fl/fl^‐Sftpc cre and *srsf5* ^fl/fl^ mice at 4dpi as determined by TCID_50_ assays. Data are from three independent experiments (*n* = 4 mice per group) run in triplicate. Error bars = SD. D) Viral NP mRNA and vRNA in lung tissues of PR8 virus‐infected *srsf5* ^fl/fl^‐Sftpc cre and *srsf5* ^fl/fl^ mice at 3 dpi as determined by RT‐qPCR. Data presented as means ± SD and are representative of three independent experiments. E) H&E staining of lung tissues from *srsf5* ^fl/fl^‐Sftpc cre and *srsf5* ^fl/fl^ mice infected with 1×10^2^ TCID_50_ of PR8 virus at 5 dpi; scale bars, 120 µm. Representative images from 4 mice per group of three independent experiments. Data presented as mean ± SD and are representative of three independent experiments. Statistical significance in A–D) was determined by unpaired two‐tailed Student's *t*‐test.

The functional relevance of SRSF5 in combating influenza virus infection in vivo was assessed in *srsf5* conditional knockout mice, since homozygous *srsf5* ^−/−^ is embryonic lethal.^[^ ^18^ ^]^ A lung‐expressing Cre line, surfactant associated protein C‐Cre (Sftpc‐Cre), was crossed with *srsf5* ^fl/fl^ mice to produce *srsf5* ^fl/fl^‐Sftpc cre mice which were genotypically confirmed by PCR (Figure S2A, Supporting Information). *srsf5* ^fl/fl^‐Sftpc cre and control *srsf5* ^fl/fl^ mice were intranasally infected with 1×10^2^ TCID_50_ of PR8 influenza virus, and their survival and body weight changes were monitored daily for 15 days. Five out of six *srsf5* ^fl/fl^ mice died by day 7 (**Figure** **2**A). In contrast, one *srsf5* ^fl/fl^‐Sftpc cre mouse died on day 7. Furthermore, *srsf5* ^fl/fl^ mice showed progressive weight loss, whereas the five surviving *srsf5* ^fl/fl^‐Sftpc cre mice started to regain body weight from 7 dpi (Figure 2B). Progeny virus production from lung tissues of infected *srsf5* ^fl/fl^‐Sftpc cre mice at 4 dpi was significantly less than from those of control mice (Figure 2C) with corresponding reduction in viral NP mRNA and vRNA (Figure 2D). Histopathology also revealed reduced in lung inflammation in *srsf5* ^fl/fl^‐Sftpc cre mice relative to *srsf5* ^fl/fl^ mice (Figure 2E). Taken together, absence of SRSF5 reduces influenza virus replication in human cells and in mice, and, in the latter, reduces severity of infection.

### 2.3 SRSF5 Increased M2 Expression from Enhanced M Pre‐mRNA Splicing

**Figure 3:**
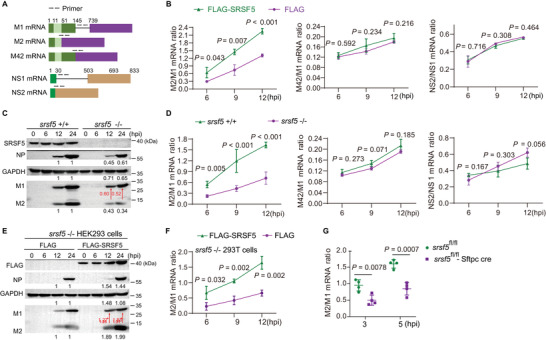
SRSF5 protein increased M2 expression from enhanced M pre‐mRNA splicing. A) M1 and NS1 mRNAs, and their alternatively spliced variants (M2, M42, and NS2) are depicted; arrowheads indicate primer positions for detecting the different transcripts. B) A549 cells were transfected with SRSF5‐Flag plasmids or empty control for 24 h and infected with PR8 virus at 1.0 MOI for 12 h. Different mRNA splicing ratios, M2/M1, M42/M1, and NS2/NS1, were derived from RT‐qPCR at 6, 9, and 12 hpi. Data presented as means ± SD. C,D) *srsf5* ^+/+^ and *srsf5* ^−/−^ HEK293 cells were infected with PR8 virus at 1.0 MOI. Protein ratios of M2/M1 were determined at 12 and 24 hpi (in red) C), and M2/M1, M42/M1, and NS2/NS1 mRNA ratios were derived from RT‐qPCR at 6, 9, and 12 hpi D). Data presented as means ± SD. E,F) *srsf5* ^−/−^ HEK293 cells were transfected with SRSF5‐Flag plasmids or empty control vector for 24 h and infected with PR8 virus at 1.0 MOI. M2/M1 protein ratios at 12 and 24 hpi (in red) E), and M2/M1 mRNA ratios at 6, 9, and 12 hpi were determined F). Data presented as means ± SD. G) *srsf5* ^fl/fl^‐Sftpc cre and *srsf5* ^fl/fl^ mice were each infected with 1 × 10^2^ TCID_50_ of PR8 virus. M2/M1 mRNA ratios were derived from lung tissues at 3 and 5 dpi by RT‐qPCR. Data are from three independent experiments with *n*  =  4 mice per group run in triplicate. Error bars indicate s.d. Data in B–G) are representative of three independent experiments. Statistical significance in B,D) and F–G) was determined by unpaired two‐tailed Student's t‐test.

SRSF5 protein is a crucial splicing factor of host genes^[^ ^19^ ^]^ but its role in IAV viral genes hitherto is unknown. Different primer pairs were designed for the specific detection of M1, M2, M42, NS1, and NS2 by RT‐qPCR (**Figure** **3**A), from which to determine the impact of SRSF5 on alternative splicing of M and NS genes. A549 cells were transfected with Flag‐SRSF5 or Flag empty vectors followed by PR8 virus infection at 1.0 MOI. Over‐expression of SRSF5 significantly increased the M2/M1 mRNA ratios at 6, 9, and 12 hpi, but had no impact on corresponding NS2/NS1 and M42/M1 mRNA ratios (Figure 3B). Similarly, over‐expression of Flag‐SRSF5 in HEK293 cells increased the M2/M1 protein ratios as determined by Western blotting (Figure S2B, Supporting Information). Conversely, infected *srsf5^−/−^* HEK293 cells clearly showed reduced M2/M1 mRNA and protein ratios in contrast to corresponding expression in *srsf5^+/+^* HEK293 cells (Figure 3C,D). No difference in the NS2/NS1 or M42/M1 mRNA ratio was found with infected *srsf5^−/−^* HEK293 (Figure 3D). Furthermore, exogenous expression of SRSF5 in srsf5^−/−^ HEK293 cells restored the ability to raise M2/M1 protein (Figure 3E) and mRNA (Figure 3F) ratios but had no impact on NS2/NS1 and M42/M1 mRNA ratios (Figure S2C, Supporting Information). Finally, M2/M1 mRNA ratios were determined from lungs of *srsf5* ^fl/fl^‐Sftpc cre and *srsf5* ^fl/fl^ mice infected with PR8 virus. At 3 and 5 dpi, M2/M1 mRNA ratios were significantly less in *srsf5* ^fl/fl^‐Sftpc cre mice relative to *srsf5* ^fl/fl^ mice (Figure 3G).

SR protein family members (SRSF1 to 7) can potentially act as NXF1 adaptors that couple pre‐mRNA processing to mRNA export.^[^ ^20^ ^]^ Nuclear and cytoplasmic M2 mRNA from *srsf5^+/+^* or *srsf5^−/−^* HEK293 cells infected with PR8 virus (MOI = 0.1) found no significant difference in M2 cytoplasmic/nuclear mRNA ratios between the two cell types (Figure S2D, Supporting Information) indicating that SRSF5 does not affect M2 mRNA export. Thus, SRSF5 specifically promotes viral M2 expression from enhanced M pre‐mRNA splicing.

### 2.4 SRSF5 via Its RRM2 Domain Directly Bound M Pre‐mRNA

**Figure 4:**
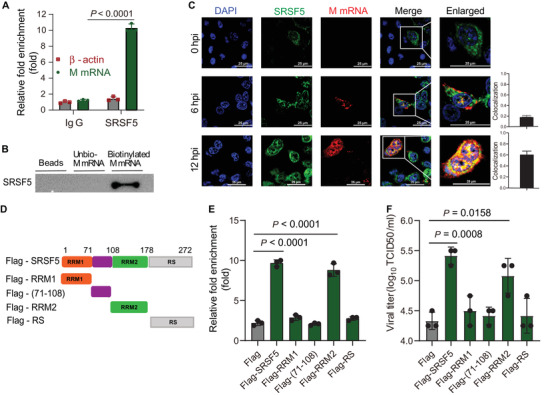
The RRM2 domain of SRSF4 directly bound M pre‐mRNA in infected cells. A) Cell lysates, from A549 cells infected with PR8 virus for 12 h, was immunoprecipitated with anti‐SRSF5 or control IgG. Bound‐RNA was extracted for qPCR analysis, with *β*‐actin mRNA detection as control. Data presented as means ± SD. B) Cell lysates were incubated with biotin‐labeled M mRNA and immunoprecipitated with streptavidin beads. Bound proteins were analyzed by immunoblots with anti‐SRSF5 antibody. Unlabeled M mRNA was used as a control. C) Colocalization of endogenous SRSF5 (green) and viral M mRNA (red) in PR8 virus‐infected A549 cells at 0, 6, and 12 hpi was demonstrated by RNA FISH. Nuclei were stained with DAPI (blue). Scale bars, 25 µm. Quantification of colocalization of SRSF5 and M mRNA in cells (right). Means ±SD from 3 biological samples. D) Human SRSF5 domains and truncation mutants generated are shown. E) A549 cells were transfected with Flag‐tagged SRSF5 plasmid, its truncated forms (as indicated) and empty vector as negative control. M mRNA eluted from Flag immunoprecipitates were quantified by RT‐qPCR. Data presented as means ± SD. F) HEK293 cells were transfected with Flag‐tagged SRSF5 (full length) plasmid and its truncated forms for 24 h and infected with PR8 virus for 18 h. Viral titers were determined by TCID_50_ assays. Data presented as means ± SD. Data in A–C) and E,F) are representative of three independent experiments. Statistical significance in A), C), and E) was determined by unpaired two‐tailed Student's *t*‐test.

Our previous study found that SRSF5 protein associates with influenza vRNA.^[^ ^17^ ^]^ Here, SRSF5 protein was found to interact with viral ribonucleoprotein (vRNP) in that all components of vRNP (PA, PB1, PB2, and NP) coimmunoprecipitated with SRSF5 (Figure S3A, Supporting Information). From cotransfection of GFP‐tagged SRSF5 plasmid with individually RFP‐tagged vRNP components (RFP‐tagged PB2, RFP‐tagged PB1, RFP‐tagged PA, or RFP‐tagged NP plasmid) in A549 cells, SRSF5 colocalized with PB2, PB1, and NP, but not with PA, in the nucleus (Figure S3B, Supporting Information). SR proteins bind RNA via two RRM domains at the N‐terminal, and proteins via the RS domain at the C‐terminal.^[^ ^12^, ^21^ ^]^ Next, RNA immunoprecipitation (RIP) with SRSF5 antibody on infected A549 cell lysates (12 hpi) was performed to detect M mRNA by RT‐qPCR. There was a 10‐fold enrichment of M mRNA relative to control *β*‐actin mRNA detection (**Figure** **4**A). Furthermore, pull‐down assay using biotinylated M mRNA on cell lysates, derived from HEK293 cells over‐expressing Flag‐tagged SRSF5, showed clear binding of SRSF5 to the M mRNA (Figure 4B). RNA fluorescence in situ hybridization (FISH) to detect endogenous SRSF5 and M mRNA showed increasing nuclear colocalization of SRSF5 with M mRNA of PR8 virus infected A549 cells over 12 h of infection (Figure 4C). In summary, SRSF5 binds and colocalizes with M mRNA in infected cells.

To determine the RNA‐binding site of SRSF5 protein, several Flag‐truncated SRSF5 expression constructs were generated for transfections in A549 cells (Figure 4D). M mRNA eluted from Flag immunoprecipitates were quantified by RT‐qPCR. Only full‐length SRSF5 and the RRM2 domain bound M mRNA in infected A549 cells (Figure 4E). Transfection of A549 cells with RRM2–GFP plasmid found strong colocalization of RRM2 protein and M mRNA at 12 hpi (Figure S3C, Supporting Information). Additionally, only full‐length SRSF5 and the RRM2 domain showed high affinity for M mRNA, as demonstrated by the use of microscale thermophoresis technology (MST) (Figure S3D, Supporting Information). Finally, SRSF5 constructs without the RRM2 domain were unable to promote IAV replication (Figure 4F). Collectively, the RRM2 domain of SRSF5 directly binds M mRNA to facilitate IAV replication.

### 2.5 M mRNA Sites at Position 163, 709, and 712 Site Was Critical for SRSF5‐Mediated AS and Viral Replication

**Figure 5:**
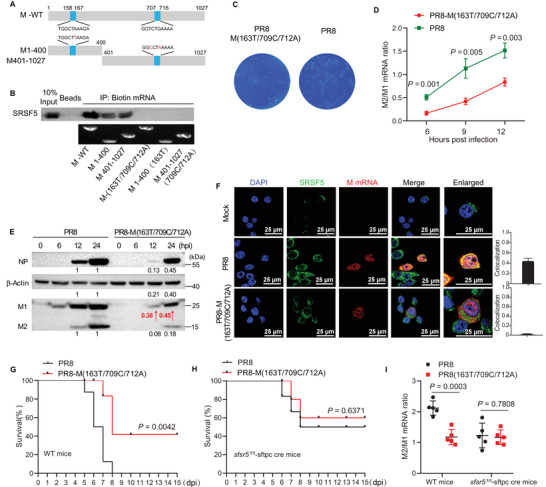
M gene mutations at position 163, 709, and 712 impaired M mRNA splicing and virus replication. A) Schematic diagram of SRSF5 binding sites in M mRNA. B) A549 cell lysates were incubated with biotinylated‐full length M mRNA or mutated M mRNAs and immunoprecipitated with streptavidin beads. Bound proteins were subjected to immuno‐detection with anti‐SRSF5 antibody. No beads were used as control. C) For plaque morphology comparison, monolayers of A549 cells were infected with PR8 virus or PR8 M(163T/709C/712A) mutated virus, overlaid with 0.5% agarose in growth medium containing 2% fetal bovine serum, and at 48 hpi stained with crystal violet. M2/M1 mRNA D) and M2/M1 protein E) ratios in A549 cells infected with PR8 virus or mutated PR8 during infection. Data presented as means ± SD. F) Colocalization of endogenous SRSF5 (green) and viral M mRNA (red) in PR8 and PR8 mutated virus‐infected A549 cells at 12 hpi (detected by RNA FISH). Nuclei were stained with DAPI (blue). Scale bars, 25 µm. Quantification of SRSF5 and M mRNA colocalization in cells (right). Means ± SD from 3 biological samples. Survival of WT mice G) and srsf5^fl/fl^‐Sftpc cre mice H) infected with 10^4^ TCID_50_ of PR8 or PR8 mutant virus (*n* = 8). Data presented were pooled from three independent experiments. Kaplan–Meier survival curves were subjected to log‐rank (Mantel–Cox) analysis. I) M2/M1 mRNA ratios in the lung of mice infected with PR8 or PR8 mutant virus at indicated time points (*n* = 5). Data in B–I) are representative of three independent experiments. Statistical significance in D) and G–I) was determined by unpaired two‐tailed Student's *t*‐test.

To determine the motif in the M pre‐mRNA that binds SRSF5 RRM2 domain, RNA eluted from Flag‐immunoprecipitation of PR8‐infected srsf5^−/−^ HEK293 cells over‐expressing RRM2‐Flag was subjected to deep sequencing analysis. Captured M RNA segments revealed two distinct binding motifs (M_158‐167_ and M_707‐716_) in M pre‐mRNA (**Figure** **5**A; and Figure S4A, Supporting Information), which were sited close to a 5’ and 3’ splice site respectively. In murine pluripotent P19 cells, SRSF5 has been shown to bind to two distinct motifs (TCTGAAR[A/G] A and TGGCTAAAR[A/C]R[A/C]),^[^ ^22^ ^]^ which show sequence homology to M_707‐716_ and M_158‐167_, respectively. Sequence alignment of the M gene from multiple influenza subtypes, including H1N1, H3N2, H7N9, H9N2, and H5N1, showed that the two binding motifs are conserved (Figure S4F, Supporting Information). To identify key site(s) within the two binding motifs, a series of mutants (without changing the amino acid code) were transcribed and tagged with biotin. Pull‐down assays using cell lysates of HEK293 cells over‐expressing Flag‐tagged SRSF5 and individual biotin‐tagged M RNA mutants indicated that M mRNA with one or both motifs were able to bind SRSF5, but mutants that housed 163T, 709C, and 712A, or all three mutations (163T/709C/712A) were not able to bind SRSF5 (Figure 5B).

To examine the functional significance of the two binding motifs in M mRNA in its own splicing and on viral replication, a combined PR8 mutant (PR8‐163T/709C/712A) was used in comparison with the wild type PR virus. PR8‐163T/709C/712A virus in A549 cells produced smaller plaques in plaque assays (Figure 5C), less infectious progeny virus at 6, 12, 24, and 36 hpi (Figure S4B, Supporting Information), and showed reduced M2/M1 mRNA ratio at 6, 9, and 12 hpi (Figure 5D) than with corresponding use of PR8 virus; however, when the latter M2/M1 ratio study was conducted on srsf5^−/−^ HEK293 cells, there was no reduction in M2/M1 mRNA ratio (Figure S4C, Supporting Information). In PR8‐163T/709C/712A virus‐infected human A549 cells, viral NP protein production and M2/M1 protein ratio were also significantly reduced compared with PR8 infected cells (Figure 5E). Accordingly, PR8‐163T/709C/712A M mRNA was less able than PR8 virus to colocalize with SRSF5 in A549 cells (Figure 5F).

We next determined the pathogenicity of the PR8 mutant virus in WT or *srsf5* ^fl/fl^‐Sftpc cre mice. PR8‐163T/709C/712A mutant virus was less pathogenic than PR8 virus in WT mice as inferred from smaller loss in body weight (Figure S4D, Supporting Information), reduced mortality (50% with mutant virus vs 100% with PR8 virus) (Figure 5G), and twofold reduction in lung viral titers at 2 and 4 dpi (Figure S4E, Supporting Information). However, reduced pathogenicity of mutant PR8‐163T/709C/712A virus relative to PR8 virus, in terms of increased survival (Figure 5H) and reduced M2/M1 lung RNA ratio (Figure 5I), was abrogated in *srsf5* ^fl/fl^‐Sftpc cre mice. Altogether, positions 163, 709, and 712 in M mRNA are critical for SRSF5 binding to increase M pre‐mRNA AS, viral replication and pathogenicity in mice.

### 2.6 SRSF5 Recruited U1 snRNP to M Pre‐mRNA by Association with U1A

**Figure 6:**
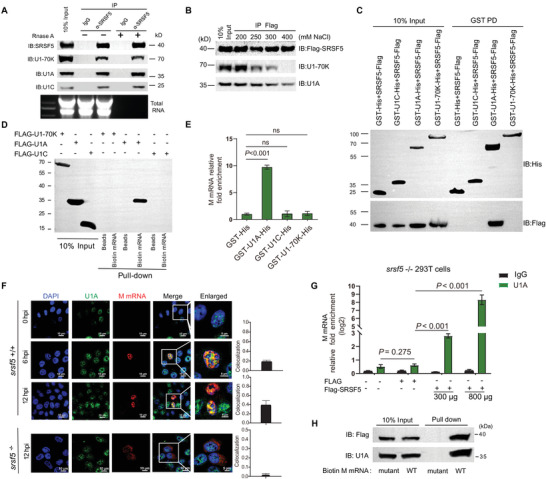
SRSF5 interacted with U1A to promote binding of U1 snRNP to M pre‐mRNA. A) A549 cells were infected with PR8 virus at 2.0 MOI for 24 h, followed by co‐IP with anti‐SRSF5 or control IgG and immunoblotting with anti‐U1‐70K, U1A, and U1C antibody in the presence or absence of RNase A. B) Association of hSRSF5‐Flag with U1 snRNP withstands high salt washes. Added SRSF5‐Flag, which was produced and purified in vitro, to cell lysates of *srsf5^−/−^* HEK293 for 1 h at 4 °C. Flag‐M2 affinity beads were added to the reaction and left for 1 h at 4 °C. The washes were done, by increasing salt concentration, from 200 to 400 mm of NaCl. Bound proteins were eluted with Laemmli and immunoblotted with antibodies directed against U1A and U1‐70K. C) SRSF5 interacts with U1A in vitro. Purified hSRSF5‐Flag was incubated with glutathione‐agarose bound GST‐U1A‐His, GST‐U1‐70K‐His, GST‐U1C‐His. Following washes, the beads were washed five times in binding buffer and the bound proteins eluted with Laemmli and immunoblotted using anti‐Flag or anti‐His antibodies. D) HEK293 cells were transfected with Flag‐tagged U1A, Flag‐tagged U1C, or Flag‐tagged U1‐70K for 24 h. Cell lysates were incubated with biotin‐labeled M mRNA and immunoprecipitated with streptavidin beads. Immuno‐detection was performed with indicated Flag antibodies. Empty beads were used as control. E) Purified GST‐U1A‐His, GST‐U1C‐His, GST‐U1‐70K‐His, or GST‐His was incubated with in vitro transcribed M mRNA for 6 h at 4 °C. M mRNA eluted from His immunoprecipitates were quantified by RT‐qPCR. Data presented as means ± SD. F) Colocalization of endogenous U1A (green) and viral M mRNA (red) in PR8 virus‐infected *srsf5* ^+/+^ and *srsf5* ^−/−^ HEK293 cells at 0, 6, and 12 hpi as detected by RNA FISH. Nuclei were stained with DAPI (blue). Quantification of colocalized SRSF5 and M mRNA in cells (right). Means ±SD from 3 biological samples. G) *srsf5* ^−/−^ HEK293 cells were transfected with increasing amount of SRSF5‐Flag expression vector or with control Flag vector, as indicated, for 24 h. Cell lysates were then immunoprecipitated with anti‐U1A or control IgG. Bound‐RNA was extracted for M mRNA detection by RT‐qPCR. Data presented as means ± SD. H) RNA pull‐down of biotinylated M mRNA or mutant M mRNA bound to U1A protein and purified Flag‐SRSF5. For A–H) data are representative of three independent experiments. Statistical significance in E) and J) was determined by unpaired two‐tailed Student's *t*‐test.

Recognition of splice sites by U1 snRNP initiates spliceosome assembly.^[^ ^23^ ^]^ SR proteins appear to facilitate U1 snRNP recognition of splice sites and regulate alternative splicing.^[^ ^24^ ^]^ To determine if SRSF5 could interact with U1 snRNP during IAV infection, HEK293 cells were infected by PR8 virus at 2.0 MOI followed by coimmunoprecipitation (Co‐IP) with anti‐SRSF5. All three core protein components of U1 snRNP (U1‐70K, U1A, and U1C) efficiently coimmunoprecipitated with endogenous SRSF5 in the presence or absence of RNase A (**Figure** **6**A). Then, HEK293 cells were cotransfected with plasmids encoding HA‐tagged SRSF5 and either Flag‐U1A, Flag‐U1C, or Flag‐U1‐70k. Co‐IP revealed that Flag‐U1A and Flag‐U1‐70K, but not Flag‐U1C, efficiently coimmunoprecipitated with HA‐SRSF5 (Figure S5A–S5C, Supporting Information). To identify which of the core components of U1 snRNP mediated direct interaction with SRSF5, we next performed a salt sensitivity test to disrupt U1 core proteins from the U1 snRNA. Flag pulldown of purified SRSF5‐Flag incubated with *srsf5^−/−^* HEK293 cell lysate were washed with buffer containing increasing salt concentration. We found that only U1A remained bound to SRSF5‐Flag at salt concentration 400 mm of NaCl, while U1‐70K dissociate from the complex at a concentration of 300–400 mm NaCl (Figure 6B). Moreover, we also incubated purified GST tagged U1A‐His, and as controls U1C‐His and U1‐70K‐His with the purified SRSF5‐Flag. Using these in vitro purified proteins, we confirmed that only GST‐U1A‐His could efficiently pulldown SRSF5‐Flag, while both GST‐U1C‐His and GST‐U1‐70K‐His as well as the negative control (GST‐His) failed to pull down SRSF5‐Flag (Figure 6C). These results suggested that the direct interaction between SRSF5 and U1 snRNP is mediated by U1A. Streptavidin pull‐down assays using cell lysates of HEK293 cells, separately over‐expressing Flag‐tagged U1A, U1C, and U1‐70K, incubated with biotinylated M mRNA revealed that only Flag‐U1A (but not Flag‐U1C and Flag‐U1‐70k) could efficiently bind to M mRNA (Figure 6D). To clarify specific interactions, the RIP‐qPCR assay was performed using the in vitro transcribed RNA and purified GST‐U1A‐His, GST‐U1C‐His, GST‐U1‐70K‐His, or GST‐His, which revealed that only GST‐U1A‐his can directly bind to M mRNA (Figure 6E). Accordingly, MST results showed only GST‐U1A‐His protein could interact with M mRNA (Figure S5D–S5F, Supporting Information). Additionally, PLA in situ analysis found increasing colocalization of SRSF5 with U1A in PR8‐virus‐infected A549 cells at 6 and 12hpi (Figure S5G, Supporting Information). Thus, SRSF5 directly interacts with U1A of U1 snRNP complex.

To determine if SRSF5 recruits U1 snRNP on M pre‐mRNA during IAV infection, the interaction between U1A and M mRNA in PR8 virus‐infected *srsf5* ^+/+^ and *srsf5* ^−/−^ HEK293 cells was examined. RNA FISH experiments in situ showed colocalization level of U1A with M mRNA in *srsf5^−/−^* HEK293 cells was much lower than that in *srsf5^+/+^* HEK293 cells (Figure 6F). Next, *srsf5* ^−/−^ HEK293 cells were transfected with increasing amount of SRSF5‐Flag expression vector or control Flag vector followed by PR8 virus infection at 1.0 MOI. RIP‐RT‐qPCR analyses showed that SRSF5 over‐expression promoted the binding of U1A to M mRNA (Figure 6G). Moreover, as baits, biotinylated full‐length M mRNA and mutant M‐163T/709C/712A mRNA were separately incubated with in vitro purified Flag‐SRSF5 protein, followed by mixing with *srsf5* ^−/−^ HEK293 cell extract. RNA pulldown found that U1A was mostly detected on the full‐length M mRNA (with SRSF5‐binding sites) (Figure 6H). These results suggest that SRSF5 association with M mRNA not only strengthens the splice site recognition by U1 snRNP, but also recruits U1 snRNP to the M mRNA leading splicing enhancement.

### 2.7 Inhibitor of SRSF5, Anidulafungin, Blocked IAV Replication

**Figure 7:**
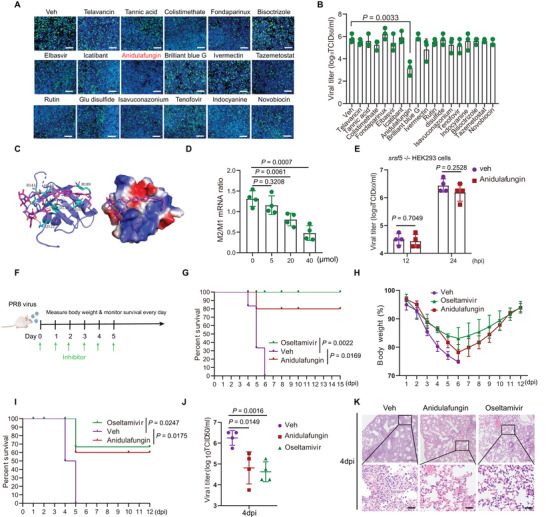
Anidulafungin targeted SRSF5 to inhibit IAV replication. A,B) A549 cells were pretreated with 17 candidate inhibitors (40 µm final concentration) 6 h before and during the infection. Candidate‐treated cells were infected with the PR8 virus at 1.0 MOI. At 24 hpi, cells were examined by fluorescence microscopy A) and infectious virus progeny in culture media was determined by TCID_50_ assays B). DMSO/DMEM was used as vehicle (Veh) control. Scale bars, 200 µm. Data in B) presented as means ± SD. C) Proposed binding interactions between anidulafungin and SRSF5 protein. D) A549 cells were treated with anidulafungin at 0, 5, 20, and 40 µm, 6 h before and during infection with PR8 virus at 1.0 MOI. At 6 hpi, splicing ratios of M mRNAs were determined by RT‐qPCR. Data presented as means ± SD. E) *srsf5* ^−/−^ HEK293 cells were treated with anidulafungin at 40 µm, 6 h before and during infection with PR8 virus at 1.0 MOI. DMSO/DMEM was used as vehicle control. At 12 and 24 hpi, viral titers were determined by TCID_50_ assays. Data presented as means ± SD. F) Schematic diagram of mouse infection design with PR8 virus and anidulafungin. G,H) BALB/c mice were infected with PR8 virus (intranasal infection, 1 × 10^3^ TCID_50_ per mouse) and orally administrated with anidulafungin (20 mg kg^−1^) or oseltamivir (20 mg kg^−1^) (*n* = 6) or DMSO/corn oil vehicle control buffer daily from day 0 to day 5. Body weight H) and survival G) were monitored daily. I,J,K) BALB/c mice were infected with PR8 virus (intranasal infection, 1 × 10^4^ TCID_50_ per mouse) and oral administrated with anidulafungin (20 mg kg^−1^) or oseltamivir (20 mg kg^−1^) (*n* = 6) or DMSO/corn oil vehicle control daily from day 0 to day 5. Survival daily monitored I). Viral titration by TCID_50_ performed on supernatants of homogenized lung tissues on day 4 (*n* =  4). J) Data presented as means ± SD. Histopathology of formalin‐fixed and hematoxylin and eosin (H&E)‐stained lung tissues on day 3 dpi K). Scale bars, 180 µm. Data in A,B), D–E), and G–K are representative of three independent experiments. Statistical significance in D,E), and G–J) was determined by unpaired two‐tailed Student's *t*‐test.

Since SRSF5, via its RRM2 domain, promoted IAV infection and replication through enhancing AS of M mRNA, a pharmacological route to target SRSF5 was explored. In the absence of a crystal structure, the 3D structure of SRSF5 was predicted by homology modeling (Figure S6A, Supporting Information). 2509 FDA‐approved drugs from its drug bank were interrogated against the RRM2 domain of SRSF5 (Figure S6B, Supporting Information). Using the Schrödinger 2013.2 software, virtual high‐throughput screening was undertaken to identify candidate SRSF5 inhibitors of which 17 were shortlisted (Figure S6C, Supporting Information). Next, each of the 17 candidates was used on A549 cells before and during infection with PR8 virus. At 24 hpi, virus infection was assessed by fluorescence microscopy of cells and progeny virus output by TCID_50_ assays. One compound, anidulafungin, a *β*‐D‐glucan synthase inhibitor approved for Candida infections treatment,^[^ ^25^ ^]^ markedly inhibited virus replication (**Figure** **7**A,B; and Figure S6D, Supporting Information). The binding mode between SRSF5 and anidulafungin is proposed (Figure 7C). Within the binding pocket of SRSF5, residues Gln122, Asp138, and Ala139 in SRSF5 (regarded as hydrogen bond acceptors) formed three hydrogen bonds with anidulafungin. Residues Arg141 and Arg189 in SRSF5 (regarded as hydrogen bond donors) formed three hydrogen bonds with anidulafungin. Residues Arg109 formed an arene‐cation interaction with anidulafungin, and residues His140 in SRSF5 formed an arene‐arene interaction with anidulafungin (Figure 7C). These interactions would have contributed to the binding energy between SRSF5 and anidulafungin.

Anidulafungin significantly reduced the ratio of M2/M1 RNA during infection in a dose‐dependent manner (Figure 7D; and Figure S6D, Supporting Information). The antiviral effect of anidulafungin in response to PR8 virus infection was absent in srsf5^−/−^ HEK293 cells, indicating that host SRSF5 is necessary to mediate its antiviral activity (Figure 7E).

Next, BABL/c mice were infected with PR8 virus (intranasal infection, 1×10^3^ TCID_50_ per mouse), followed by oral administration of anidulafungin (20 mg kg^−1^) or oseltamivir (20 mg kg^−1^) daily starting at day 0 (Figure 7F). All control mice (DMSO/corn oil treatment) died or exceeded 25% weight between 4 and 6 dpi (Figure 7G,H). In contrast, anidulafungin and oseltamivir treated groups showed prolonged survival, with 80% of the anidulafungin‐treated mice gaining body weight from day 6 of infection (Figure 7G,H). With a higher PR8 virus infection dose of 1×10^4^ TCID_50_, both anidulafungin and oseltamivir groups showed similar survival of 50–60% death at 5 dpi (Figure 7I). Both anidulafungin and oseltamivir treated mice produced significantly less infectious progeny from lung tissues (Figure 7J), and less severe lung pathology than those of control mice (Figure 7K). In summary, anidulafungin appears effective as an antiviral against lethal influenza virus challenge in mice with reduced progeny virus production and lung pathology.

## 3 Discussion

In the present study, we demonstrated that IAV‐induced SRSF5 up‐regulated the splicing of M RNA to increase M2 expression, and enhanced virus replication and pathogenicity in human cells and mice. Mechanistically, SRSF5, via its RRM2 domain, binds M pre‐mRNA, and recruits U1 snRNP by interacting with U1A to initiate and enhance M pre‐mRNA splicing.

SRSF5 knockdown or knockout significantly inhibited influenza virus replication which is consistent with an earlier finding that SRSF5 knockdown impairs the replication of influenza WSN/H1N1 virus.^[^ ^26^ ^]^ However, SRSF5 over‐expression in separate cotransfections with NS genes from H1N1, H5N1, and H3N2 virus had little effect on NS RNA splicing with H1N1 segment, or inhibited NS RNA splicing with H5N1 and H3N2 segments;^[^ ^27^ ^]^ these findings could be peculiar to NS gene but would need to be conducted with corresponding live virus for confirmation. There is strong evidence to indicate that transcription, splicing and nuclear export are often coupled to ensure coordinated control of viral gene expression.^[^ ^28^ ^]^ Given that SRSF5 can bind influenza vRNP complex (Figure S3A,S3B, Supporting Information), viral polymerase complex could also be important for viral gene splicing. Further studies are needed to evaluate the contribution of viral polymerase to SRSF5‐mediated splicing of viral genes in different influenza subtypes.

Viral infections can induce the expression of host genes that promote viral replication. In the present study, IAV infection up‐regulated the mRNA and protein expression of *SRSF5*; in turn, SRSF5 promoted IAV replication in vitro and in mice. We recently reported that PR8 virus infection also raises mRNA levels of *SRSF1*, *SRSF2*, *SRSF3*, *SRSF5*, *SRSF6*, and *SRSF7* in THP cells.^[^ ^17^ ^]^ SARS‐CoV‐2 infection transcriptionally up‐regulates *SRSF5* in human cells.^[^ ^29^ ^]^ In lungs of mice infected with H7N9 or H9N2 virus, SRSF5 production was elevated.^[^ ^30^ ^]^ In addition, tumor can induce SRSF5 expression by increasing Tip60‐mediated acetylation of SRSF5 to inhibit degradation.^[^ ^19^ ^]^ Furthermore, influenza virus can co‐opt host hnRNP K to promote infection directly by inducing viral gene splicing, and indirectly by mediating splicing of pro‐viral transcripts.^[^ ^31^ ^]^ Thus, in IAV infection additional mechanisms could be involved in alternative post‐transcriptional splicing of viral genes or post‐translational modification of SRSF5.

Influenza viral pre‐mRNA contains splice sites whose sequences are highly similar to human consensus splice sites. A purine‐rich splicing enhancer sequence, recognized by splicing factor SRSF1, has been located in the 3′ exon of M1 mRNA that promotes alternative splicing in M2 mRNA formation.^[^ ^32^ ^]^ The diversity of splice sites sequences located in influenza virus genes could contribute to different viral phenotypes.^[^ ^33^ ^]^ Several circulating avian influenza viruses (AIVs), including H9N2 and H7N9 viruses, carry point mutations in the splice site of NS segment that confer enhanced virus replication in mammalian cells,^[^ ^34^ ^]^ indicating a possible route for cross‐species virus transmission. In this study, our RNA‐seq and sequence alignment analysis revealed that multiple subtypes of AIV and human influenza virus contain the two conserved SRSF5‐binding motifs near the 5’ and 3’ splice sites of M pre‐mRNA. Mutations at M‐163/709/712, where the two motifs are sited, significantly reduced PR8 virus replication in vitro and in vivo. Thus, our study has also uncovered a possible splicing‐related mechanism that could facilitate occasional AIV infections in humans.

Alternative splicing of viral NS pre‐mRNA is adversely affected in cells depleted of RED or SMU1, leading to reduced production of spliced mRNA species NS2, and reduced NS2/NS1 protein ratio.^[^ ^35^ ^]^ Recently, human factor TRA2A was found to inhibit avian IAV replication, but benefits human IAV replication by altered regulation of viral mRNA splicing, which further suggests that alternative viral mRNA splicing is crucial for virus adaptation in humans.^[^ ^8c^ ^]^ Host proteins NS1‐BP and hnRNP K, which bind M mRNA downstream of M2 5’ splice site, increase splicing of M mRNA and promote replication of IAV.^[^ ^36^ ^]^ Subsequent research revealed that NS1‐BP binds M mRNA most proximal to the 5′ splice site, partially overlapping the U1 snRNP binding site, while hnRNP K binds further downstream.^[^ ^9b^ ^]^ We demonstrated in vitro and in vivo that SRSF5 protein promotes alternative splicing of IAV M pre‐mRNA by recruiting U1 snRNP (through interacting with U1A) to the splice sites, which leads to increase in M2 protein production and virus replication. However, besides M mRNA splicing, SRSF5 may have a wider functional role in regulating the splicing of host antiviral genes.

Exploration of host factors involved in the splicing of viral genes provides a basis for developing alternative host‐directed antiviral drugs. Two synthetic molecules that interfered with RED–SMU1 complex assembly have been shown to have antiviral potential by inhibiting the splicing of NS mRNA.^[^ ^3c^ ^]^ Alternative splicing of mRNA can alter the phenotypic diversity in many types of cancers.^[^ ^37^ ^]^ For example, SRSF6 mediates colorectal cancer progression through regulating alternative splicing, and indacaterol, a licensed bronchodilator drug, through targeting SRSF6, has been repositioned for antitumor use.^[^ ^38^ ^]^ In the present study, anidulafungin, a licensed antifungal agent, has been identified as a potential antiviral, based on clear supporting evidence of in vitro and in vivo virus inhibition, via its ability to bind to the RRM2 domain of SFSR5. Anidulafungin could suppress the infection and replication of Zika virus^[^ ^39^ ^]^ and HIV^[^ ^40^ ^]^ in vitro, suggesting that the functional relationship between SRSF5 and viral gene splicing may also be present in different viruses. Work is needed to evaluate the efficacy of anidulafungin against other influenza subtypes.

In summary, we highlighted the functional importance of SRSF5 in promoting IAV replication through the up‐regulation of M2 derived from alternative splicing of M mRNA, and provided strong preclinical evidence for repositioning anidulafungin as an anti‐influenza drug that targets SRSF5.

## 4 Experimental Section

### Mice

Lung‐specific *srsf5*‐deficient (*srsf5* ^fl/fl^‐Sftpc cre) mice and *srsf5* ^fl/fl^ mice were purchased from the GemPharmatech (Nanjing, China) and bred in Biosafety Level II laboratory (BSL‐2). Wild type (WT) C57BL/6J mice were purchased from Laboratory Animal Technology of Charles River, Beijing. Mice were maintained in specific pathogen‐free conditions, and were used in experiments at between 6 and 8 weeks of age. All mice experiments were performed in accordance with institutional guidelines of China Agricultural University (CAU) (approval SKLAB‐B‐2010‐003) and approved by the Beijing Association for Science and Technology of China (approval SYXK, Beijing, 2007–0023).

### Cells and Viruses

Human lung adenocarcinoma epithelial cells (A549), Madin–Darby canine kidney cells (MDCK) and human embryonic kidney cells (HEK293) were maintained in the laboratory. *Srsf5* ^−/−^ HEK293 cell line (ab266246) were from Abcam. Cells were cultured in DMEM supplemented with 10% v/v heat‐inactivated fetal bovine serum (Gibco), 100 U mL^−1^ penicillin and 100 µg mL^−1^ streptomycin. Influenza A/Puerto Rico/8/1934 (PR8, H1N1), A/chicken/Hebei/2008 (H9N2), and A/Anhui/1/2005 (H5N1) viruses were maintained in the laboratory. GFP‐tagged PR8 virus was generated by inserting the GFP coding sequence at the carboxyl terminal of NS1 as previously described.^[^ ^17^ ^]^

### Plasmid Construction and Transfection

*srsf5*, *U1A*, *U1C*, and *U1‐70K* genes were amplified by PCR using A549 cells and expression constructs were generated using PRK5 containing different tags or pCDNA3.1–GFP vectors by recombinase‐mediated recombination. Plasmid transfections in HEK293 or A549 cells were performed using Lipofectamine 3000 reagent (Invitrogen).

### Antibodies and Reagents

Antibody concentrations for immunoprecipitation and immunoblotting were determined empirically. All immunoblot antibodies were diluted as specified in 5% w/v BSA‐TBST. Antibodies used as follows: SRSF5 (Abcam, ab67175), SRSF5 (Thermo Fisher, PA560383), U1A (Santa Cruz Biotechnology, sc‐376027), U1C (Santa Cruz Biotechnology, sc‐101549), U1‐70K (Santa Cruz Biotechnology, sc‐390899), Tubulin (Abcam, ab6046), FLAG (Abcam, ab205606), GAPDH (California Bioscience, CB100127), influenza virus M1 (GeneTex, GTX125928), M2 (GeneTex, GTX125951), PA (GeneTex, GTX118991), PB1 (GeneTex, GTX125923), PB2 (GeneTex, GTX125926), NP (GeneTex, GTX125989), control rabbit IgG polyclonal (ABclonal Biotechnology, AC005), Fluor 488‐goat anti‐rabbit IgG (Abcam ab150077), Fluor 647‐goat anti‐rabbit (Abcam, ab150079), Fluor 488‐goat anti‐mouse IgG (Abcam ab150113) and Fluor 647‐goat anti‐mouse (Abcam, ab150115).

The 17 drugs selected by virtual screening were purchased from MCE: telavancin (HY‐16485), tannic acid (HY‐B2136), colistimethate (HY‐A0214), fondaparinux (HY‐B0597), elbasvir (HY‐15789), icatibant (HY‐108896), anidulafungin (HY‐13553), brilliant blue G (HY‐D0014), ivermectin (HY‐15310), rutin (HY‐W013075), glutathione disulfide (HY‐D0844), isavuconazonium (HY‐100373), tenofovir disoproxil (HY‐13910), indocyanine green acid form (HY‐D0711), bisoctrizole (HY‐B0897), tazemetostat (HY‐13803C), and novobiocin (HY‐B0425A).

### Mice Infection

Mice were anesthetized and intranasally inoculated with PR8 virus at a median tissue culture infectious dose (TCID_50_) of 100 in 50 µL of PBS. Body weight and survival were monitored daily. Lung tissue lysates were generated by homogenizing snap‐frozen lung tissues twice (20 s each time) in MEM medium and centrifuging the lung suspensions at 2000 rpm for 15 min. TCID_50_ assays were performed using MDCK cells and TCID_50_ was calculated as previously described.^[^ ^41^ ^]^

### Histology

*srsf5* ^fl/fl^‐Sftpc cre, *srsf5* ^fl/fl^, and WT mice were euthanized at the indicated time points postinfection. Lung tissues were collected and fixed with 4% paraformaldehyde, followed by paraffin embedding. For histopathological analysis, 5–7 µm sections were sectioned longitudinally through the left and right lung and stained using a standard haematoxylin and eosin (H&E) protocol.

### In Vitro Transcription and Biotin‐Labeling RNA Purification

Templates for T7 RNA transcription were linearized from H1N1 pSPT9 plasmids coding for individual RNA segments of PR8/H1N1 virus. T7 transcription reactions were carried out with T7 RNA polymerase in transcription buffer and biotin‐dNTPs mix according to the manufacturer's instructions (Promega). Following DNase I treatment, biotin‐labeled mRNAs were extracted with phenol–chloroform, ethanol precipitated and purified with RNAeasy columns (Aidlab Biotechnologies) and analysed on denaturing agarose gels for correct size.

### RT‐qPCR

Total RNA from virus‐infected cells or lung tissues was extracted using an RNA isolation kit (Thermo Scientific). First‐strand cDNA was synthesized from 1 µg of total RNA using a TransScript RT reagent kit (TransGen). Uni‐12 primer was used for the detection of influenza vRNA, and oligo dT and random primers were used for detecting host and viral genes. Generated cDNA was subjected to qPCR in a 20 µL reaction volume using FastStart Universal SYBR Green master mix (Roche). Human *β*‐actin and mouse GAPDH genes were amplified for normalization in qPCR. Reactions were conducted in triplicates, and the data were analysed using the 2−ΔΔCt method. qPCR primers used in this study are provided in the Table S1 (Supporting Information).

### Coimmunoprecipitation (Co‐IP)

A549 cells were infected with 1.0 MOI of PR8 virus. Postinfection, cell samples were collected and lysed in 800 µL of IP lysis buffer (Thermo Scientific) containing protease and phosphatase inhibitors. A portion of each whole cell lysate sample was kept to confirm protein expression levels, and 500 µg of cell lysate was used for the co‐IP. Lysates were incubated with anti‐SRSF5 and anti‐U1A antibodies overnight at 4 °C under constant rotation, and then 40 µL of protein A/G beads (Santa Cruz) were added and incubated for 2 h at 4 °C under gentle rotation. The beads were then washed four times with cold lysis buffer and analyzed by SDS–PAGE and Western blotting.

### RNA FISH

A549 cells were grown in 24‐well slide chambers and infected with PR8 at 1.0 MOI. At 12 hpi, cells were fixed for 15 min in 4% paraformaldehyde, permeabilized, and dehydrated by sequential 3 min incubations as follows: once with 70% ethanol, once with 85% ethanol and three times with 100% ethanol. Cells were then hybridized with Alexa Fluor 488‐conjugated M RNA target probes (M‐probes, GenePharma) of PR8 virus in hybridization buffer for 10 min at 75 °C. Cells were further incubated for 12–16 h at 37 °C. Finally, cells were stained with anti‐SRSF5 and secondary antibodies, and nuclei were stained with 4,6‐diamidino‐2‐phenylindole (DAPI) as previously described.^[^ ^17^ ^]^

### Microscale Thermophoresis Technology (MST)

HEK293 cells were separately transfected with the SRSF5‐GFP, RRM1‐GFP, RRM2‐GFP, and RS‐GFP expression vectors. After 24 h of transfection, cell lysates were collected and incubated with twofold serial dilutions of indicated mRNAs in MST‐optimized buffer at a constant concentration (20–100 nm). Equal volumes of binding reactions were mixed by pipetting and incubated for 15 min at room temperature. Mixtures were enclosed in standard‐treated glass capillaries and loaded into the instrument (Monolith NT.115, NanoTemper). Dissociation constant (Kd) values were determined using the NanoTemper analysis tool.

### RNA Pull‐Down Assay

HEK293 cells were transfected with Flag‐tagged SRSF5 vectors. After 24 h of transfection, cells were collected and lysed with lysis buffer (50 mm Tris‐HCl pH 7.0, 150 mm NaCl, 1 mm MgCl_2_ and 0.05% NP‐40). Cell lysates were mixed with transcribed biotin‐labeled viral M mRNA for 4 h at 4 °C and incubated with prewashed Dynabeads M‐280 Streptavidin (Sigma) for another 3 h at 4 °C. The protein samples bound to the beads were boiled and analyzed by SDS–PAGE and Western blotting.

### In Situ Proximity Ligation Assay (PLA) Microscopy

A DuoLink PLA kit (DUO92105‐1KT, Sigma) was used to test protein–protein interactions as described in the protocol. A549 cells were infected with PR8 virus at 1.0 MOI for 12 h, fixed and permeabilized, as described in the confocal microscopy section, and blocked with DuoLink blocking buffer for 30 min at 37 °C. Cells were incubated with corresponding primary antibodies diluted in DuoLink dilution buffer. After washing, cells were incubated with species‐specific PLA probes (plus and minus) for 1 h at 37 °C under hybridization conditions and in the presence of two additional oligonucleotides to facilitate hybridization of PLA probes if they were in close proximity (< 40 nm). Ligase was then added and incubated for 30 min at 37 °C to ligate hybridized oligonucleotides. Amplification polymerase was added to generate a concatemeric product extending from the oligonucleotide arm of the PLA probe. Finally, a detection solution consisting of fluorescence‐labeled oligonucleotides was added, and the labeled oligonucleotides were hybridized to the concatemeric products. Nuclei was stained with Duolink in situ mounting medium containing DAPI.

### Confocal Microscopy

A549 cells on coverslips were washed twice with prewarmed PBS and fixed with 4% paraformaldehyde for 15 min at room temperature. Cells were subsequently permeabilized with immunostaining permeabilization buffer containing Triton X‐100 (Beyotime) for 10 min and blocked with QuickBlock blocking buffer for 20 min at room temperature. Fixed cells were incubated with indicated antibodies diluted in immunostaining primary antibody dilution buffer at 4 °C overnight. Coverslips were then washed three times with PBS and incubated with Alexa Fluor 488‐conjugated secondary antibodies or Alexa Fluor 555‐conjugated secondary antibodies for 1 h at 37 °C. Coverslips were finally washed three times and mounted onto microscope slides with DAPI staining solution (Beyotime) for 8 min and examined by confocal microscopy. Immunostained cells were visualized using a Nikon super‐resolution laser scanning confocal microscope under a 100‐time oil objective and analyzed using the Imaris 9.2 platform.

### RNA Immunoprecipitation (RIP)‐qPCR

The RIP‐qPCR assay was performed as previously described.^[^ ^42^ ^]^ Two 10 cm^2^ dishes (10^7^ cells per dish) of A549 cells were infected with PR8 virus for 12 h. Cells were lysed with RIP lysis buffer (50 mm HEPES, 150 mm KCl, 2 mm EDTA, 1 mm NaF, 0.5% NP40, 0.5 mm dithiothreitol (DTT), 1× protease inhibitor cocktail and 25U RNasin) for 30 min at 4 °C. Cell lysates were centrifuged at 12 000 rpm for 15 min at 4 °C and the supernatants were subjected to RIP. A 50 µL aliquot of cell supernatant was saved as input, and the remaining samples were each incubated with 5 µg anti‐SRSF5 antibody or IgG antibody and 40 µL protein A/G beads for 10 h at 4 °C under gentle shaking. After IP, the beads were pelleted and washed four times with RIP wash buffer (50 mm Tris pH 7.4, 150 mm NaCl, 1 mm MgCl_2_, and 0.05% NP40), resuspended in 250 µL of DNase digestion buffer (40 mm Tris pH 8.0, 10 mm MgSO_4_, and 1 mm CaCl_2_) and treated with 25U RNasin (Promega) and 2U DNase I (NEB) at 37 °C for 20 min. Beads were then washed and resuspended in 100 µL RIP wash buffer, 10% of each sample was removed for immunoblot analysis. Samples were treated with 4U proteinase K at 55 °C for 30 min. The input and immunoprecipitated RNAs were isolated with 1 mL of TRIzol reagent (Sigma), and vRNAs were analyzed by RT–qPCR or RNA‐seq.

### Protein Purification and GST Pulldown

Competent *E. coli* BL21‐CodonPlus (DE3)‐RIPL strain were transformed with pEN‐GST‐SRSF5‐flag, pEN‐GST‐U1A‐His, pEN‐GST‐U1–70k‐His, pEN‐GST‐U1C‐His and empty vectors. Single colony of each construct was then grown in LB media at 37 °C until desired density, and then induced with 0.3 mm IPTG (Beyotime Biotechnology, #ST098) at 30 °C overnight. GST‐tagged proteins were purified using standard protocols with the GST‐tag Protein Purification Kit (Beyotime Biotechnology, #P2262) according to the manufacturer's instructions. For GST‐SRSF5‐flag, the GST‐tag was removed by 20 units of PreScission Protease (2 U µL^−1^; Beyotime Biotechnology, #P2302) at 4 °C overnight in 50 mm Tris‐HCl, 150 mm NaCl, 1 mm EDTA, 1 mm DTT, pH7.5. For GST pulldown, 300 ng of purified recombinant SRSF5‐Flag was incubated with 150 ng of glutathione bound GST‐tagged U1A‐His, U1‐10K‐His, U1‐C‐His or with negative control GST‐His in binding buffer (50 mm Tris, pH 7.5, 200 mm NaCl, 10% glycerol, 0.5% Triton‐X‐100, 10 mg mL^−1^ RNaseA), supplemented with 1× protease inhibitor complete EDTA‐free (Beyotime Biotechnology). The reaction volume was made upto 300 µL in total and incubated for 1 h at 4 °C. Beads were washed and bound proteins were resolved by SDS‐PAGE and analyzed by western blot.

### Cell Viability Assay

A549 cells or HEK293 cells were plated in 96‐well plates at a density of 800 cells in 100 µL of medium per well 24 h before the experiment. Following treatments, the cell viability was analyzed using a CCK8 kit (Cell Counting Kit‐8).

### Inhibitors Use In Vivo

All animal procedures (including infection, weighing, and killing) were performed under anesthesia with zoletil. Mice were intranasally infected with 10^3^ TCID_50_ of PR8 virus in 50 µL PBS. Anidulafungin or oseltamivir was first dissolved in DMSO and diluted in corn oil for oral administration. The same volume of DMSO/corn oil was used in vehicle groups. Anidulafungin or oseltamivir was orally administrated daily (20 mg kg^−1^ body weight) from the indicated starting time points to 5 dpi. On day 4, a subset of mice was killed by isoflurane overdose and tissue samples were collected for histopathology and viral titer analyses. The left lung of each mouse was fixed by formalin for sectioning and H&E staining. The right lung was weighed and stored at −80 °C until being homogenized in 1 mL DMEM and titrated by TCID_50_ assay.

### Homology Modeling

Human SRSF5 protein sequence Uniprot ID is Q13243. Template crystal structure was identified through NCBI BLAST and downloaded from RCSB Protein Data Bank (http://www.rcsb.org/) and the PDB ID is 2M8D1. Homology modeling was conducted in MOE v2018.012. The protonation state of the protein and the orientation of the hydrogens were optimized by LigX, at the pH 7 and temperature of 300 K. First, the target sequence was aligned to the template sequence, and ten independent intermediate models were built. These different homology models were the result of the permutational selection of different loop candidates and side chain rotamers. The intermediate model which scored best according to the GB/VI scoring function was chosen as the final model. The final model was subjected to further energy minimization using the AMBER10: EHT force field.

### Virtual Drug Screening

The dock module in MOE v2018.01 was used for structure‐based virtual screening (SBVS). The predicted structure of SRSF5 was defined as receptor. The 2509 FDA‐approved drugs structures downloaded from DrugBank (https://go.drugbank.com/) were selected as virtual screening library. All compounds were prepared with the Wash module in MOE. The protonation state of the protein and the orientation of the hydrogens were optimized by QuickPrep module at pH 7 and temperature of 300 K. All compounds were first ranked by high throughput rigid docking with London dG scoring. Prior to docking, the force field of AMBER10: EHT. For flexible docking, docked poses were ranked by London dG scoring first, then a force field refinement was carried out on the top 10 poses followed by a rescoring of GBVI/WSA dG and the best ranked pose was retained. After flexible docking, the top ranked 100 compounds were divided into 50 structural clusters through fingerprint‐based clustering. The best ranked 50 cluster centers were finally identified as potential hits.

### Statistical Analysis

For all the bar graphs, data are shown as the mean ± SD. All statistical analyses were performed using GraphPad Prism software v.8.00 (GraphPad Software). Unpaired Student's *t*‐test was used to perform statistical analysis between the two groups. The Kaplan–Meier method was employed for survival analysis. Differences in means were considered statistically significant at *P* < 0.05; significance levels are as follow: *P < 0.05; **P < 0.01; ***P < 0.001; NS, not significant.

## Conflict of Interest

The authors declare no conflict of interest.

## Supporting information

Supporting Information

## Data Availability

The data that support the findings of this study are available from the corresponding author upon reasonable request.
