# Supplementary material for: SRSF5‐Mediated Alternative Splicing of M Gene is Essential for Influenza A Virus Replication: A Host‐Directed Target Against Influenza Virus
Source: Adv Sci (Weinh). 2022 Oct 18;9(34):2203088. doi: 10.1002/advs.202203088 (PMC9731694; doi:10.1002/advs.202203088)
Supplement: Supplementary file 1 — Supporting Information [file ADVS-9-2203088-s001.pdf]

ADVANCED SCIENCE Open Access

Supporting Information

for *Adv. Sci.*, DOI 10.1002/advs.202203088

SRSF5-Mediated Alternative Splicing of M Gene is Essential for Influenza A Virus Replication: A Host-Directed Target Against Influenza Virus

*Qiuchen Li, Zhimin Jiang, Shuning Ren, Hui Guo, Zhimin Song, Saini Chen, Xintao Gao, Fanfeng Meng, Junda Zhu, Litao Liu, Qi Tong, Honglei Sun, Yipeng Sun, Juan Pu, Kin-Chow Chang and Jinhua Liu**

Supporting Information

SRSF5-mediated alternative splicing of M gene is essential for influenza A virus replication: a host-directed target against influenza virus

Qiuchen Li,[§] Zhimin Jiang,[*] Shuning Ren,[§] Hui Guo, Zhimin Song, Saini Chen, Xintao Gao, Fanfeng Meng, Junda Zhu, Litao Liu, Qi Tong, Honglei Sun, Yipeng Sun, Juan Pu, Kin-Chow Chang, Jinhua Liu[*]

Dr Q. C. Li, Dr Z. M. Jiang, Dr S.N.Ren, S. N. Chen, Dr F. F. Meng, Dr J. D. Zhu, Dr L. T. Liu, Dr Q. Tong, Prof. H. L. Sun, Prof. Y. P. Sun, Prof. J. P, Prof. J. H. Liu
Key Laboratory for Prevention and Control of Avian Influenza and Other Major Poultry Diseases, Key Laboratory of Animal Epidemiology, Ministry of Agriculture, College of Veterinary Medicine, China Agricultural University, Beijing 100193, China
E-mail: ljh@cau.edu.cn (J. L.)

Dr Z. M. Jiang, Dr H. Guo, Dr Z. M. Song
Chinese Academy of Sciences Key Laboratory of Infection and Immunity, Institute of Biophysics, Chinese Academy of Sciences, Beijing 100101, China

Dr. X. T. Gao
Biotechnology Research Institute, Chinese Academy of Agricultural Sciences, Beijing 100081, China

Prof. K. C. Chang
School of Veterinary Medicine and Science, University of Nottingham, Sutton Bonington Campus, Sutton Bonington LE12, UK.

[§]These authors contributed equally to this work.

Table S1. List of primer pairs used for real-time PCR in this study.

Gene	Forward (5'-3')	Reverse (5'-3')
	**Human Gene**	
Srsf1	TATCCGCGACATCGACCTCAAG	AAACTCCACCCGCAGACGGTAC
Srsf2	GCGGTGGCTACGGACGCCG	TTCGAGCGGCTGTAGCGAGATC
Srsf3	GATTATCGTAGGAGGAGTCCTCC	ACGGCTTGTGATTTCTCTCCCG
Srsf4	CAGATTAGTTGAAGACAAGCCAGG	CACTTCGGCTTCTGCTCTTACG
Srsf5	GGTGGTTGAGTTTGCCTCTTATG	GATCGAGACCTGCTTCTTGACC
Srsf6	GTGCTTTGGACAAACTGGATGGC	CTCCTACTTCGTGACCGTCTTC
Srsf7	CTATGAGTGTGGCGAAAAGGGAC	GAGTATCGCCTTCCTCTGGATC
Srsf8	TCGAAGTCTGGGTCCTCCACTA	GGAGGACTCCTGGTCATAGATG
Srsf9	CCTGCGTAAACTGGATGACACC	CCTGCTTTGGTATGGAGAGTCAC
Srsf10	GGAGGAGATCAAGAAGTCGGTC	TGTCGGAATGGCTTCTGCTACG
Srsf11	TCCAGACTCAGCAGTTGTGGCA	GCATTAGCTGGTGCCAACAGAG
Srsf12	GAAATAGGAGGCGGTCAGACAG	TGACTGCCTTGCTGAGGTAGAC
β-actin	CCGCGAGAAGATGACCC AGAT	CGTTGGCACAGCCTGGATAGCAA
Srsf5-shRNA	GGAUCCAAGGGAUGCAGAUTT	AUCUGCAUCCCUUGGAUCCTT
	**Mouse Gene**	
Srsf5	TTTGAGGACCCAAGGGATGCAG	AAACGGTCGGAGTATCGTCCTC
flox (p1)	ATTTGTCACGTCCTGCACGA	ACCAACTCTACTAACAAGGTCGCC
flox (p2)	GGGTGCTAGGAATCAAACTCCAG	CTACTCACCAATGAGTACGCCTG
Sftpc-ki (p3)	ACCTTGTGAATGACCTCCAGG	CATATAGACAAACGCACACCGGC
Sftpc-wt (p4)	TGCTTCACAGGGTCGGTAGAAAC	TAACACCCGTGTATGGCACCC
GAPDH	ACAACTTTGGCATTGTGGAA	GATGCAGGGATGATGTTCTG
	**Influenza A virus**	
M1	ATCAGACATGAGAACAGAATGG	TGCCTAGCCTGACTAGCAACCTC
M2	CGAGGTCGAAACGCCTATCAGAAAC	CCAATGATATTTGCGGCAATAGCGAG
M42	ACCGATCTTGAGGCCTATCAGAAAC	CCAATGATA TTTGCTGCAATGACGAG
NS1	TGGAAAGCAGATA GTGGAGCG	GTAACGCGACGCAGGTACAGAG
NS2	CTTCTCCAAGCGAATCTCTGTAGA	CTTCTCCAAGCGAATCTCTGTAGA
NP	AGGGTCGGTTGCTCACAAGTC	TTGAAGCAGTCTGAAAGGGTCTA
M pre-mRNA	CATGGAGGTTGCTAGTCAGGC	ATATTTGCGGCAATAGCGAGA
Uni12	AGCAAAAGCAGG	AGTAGAAACAAGG

Supplementary Figure Legends

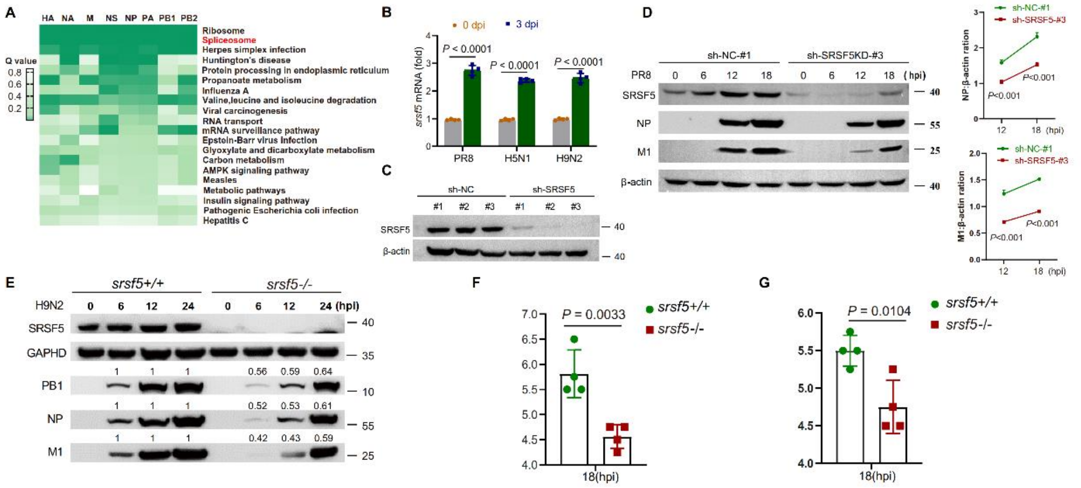

Figure S1. SRSF5 knockdown inhibited IAV replication. **A**, Protein domain types significantly enriched among the human interacting proteins with indicated RNA baits. The value range of Q value is [0.1]. The closer Q is to zero, the most significant the enrichment. Domains with Q under 0.05 are defined as domains that are significantly enriched in differentially expressed genes. **B**, *SRSF5* mRNA detection in the lung homogenates of mice challenged with H5N1 (1×10^3 $TCID_{50}$), PR8 (1×10^3 $TCID_{50}$) or H9N2 (1×10^4 $TCID_{50}$) viruses at 0 dpi and 3 dpi was determined by RT-qPCR; n = 4 for each group. Data were analyzed by an unpaired Student's t-test and shown as means ± SD. Data are representative of at least three independent experiments. **C**, A549 cell were transfected with SRSF5-targeting shRNA#1, shRNA#2, shRNA#3 or negative controls (NC) shRNA#1, shRNA#2, shRNA#3 for 24 h followed by SRSF5 protein detection by Western blotting. **D**, A549 cell were transfected with SRSF5-targeting shRNA#3 and negative control (NC) shNC#1 for 24 h, followed by infection with PR8 virus (MOI = 1). NP and M1 protein expression were detected by Western blotting at 0, 6, 12 and 18 hpi; β-actin detection was used as loading control. **E**, Time course viral NP, M1 and PB1 protein detection from H9N2 virus-infected srsf5$^{+/+}$ and srsf5$^{-/-}$ HEK293 cells at 1.0 MOI; β-actin detection was

used as a loading control. **F**, **G**, Viral titers from H9N2 **(D)** or H5N1 **(E)** virus-infected (at 1.0 MOI) srsf5$^{+/+}$ and srsf5$^{-/-}$ HEK293 cells were determined by $TCID_{50}$ assays at 18 hpi. Data presented as means ± SD and are representative of three independent experiments. Data **B**-**G** are representative of three independent experiments. Statistical significance in **D**, **F** and **G** was determined by unpaired two-tailed Student's t-test.

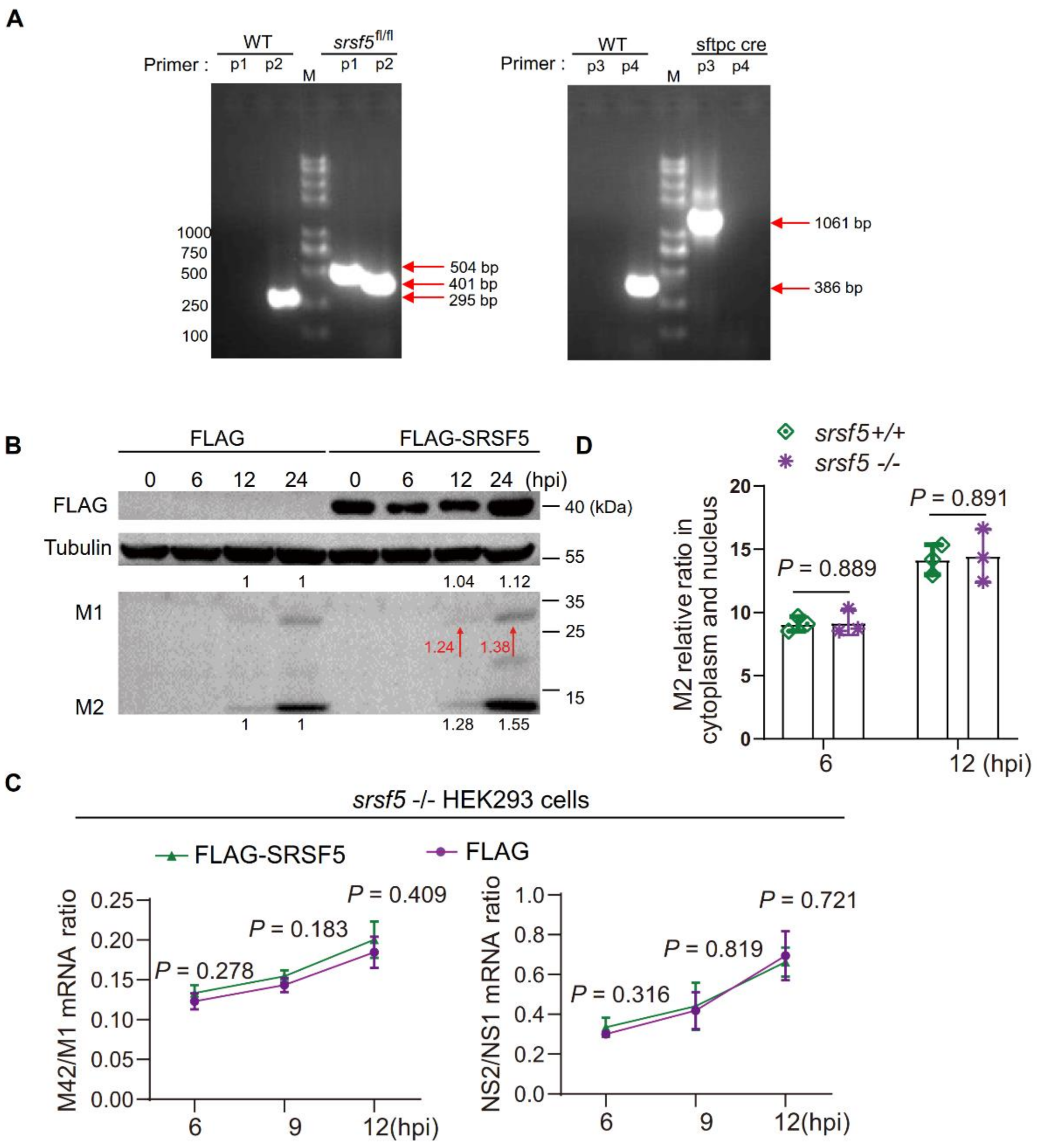

Figure S2. SRSF5 increased splicing of viral M pre-mRNA. A. Genotypes of WT and *srsf5* $^{fl/fl}$-sftpc cre mice confirmed by PCR. Left panel, WT: p1 PCR yielded no band, p2 PCR yielded a 295bp band; homozygous *srsf5*$^{fl/fl}$: p1 PCR yielded a 504bp

band, p2 PCR yielded a 401bp band. Right panel, WT: p3 PCR yielded no band; p4 PCR yielded a 386bp band; homozygous sftpc cre: p3 PCR yielded a1061bp band; p4 PCR yielded no band. **B**, A549 cells were transfected with SRSF5-Flag plasmids or empty control for 24 h and infected with PR8 virus at 1.0 MOI. M2/M1 protein ratios were derived from Western blotting at 0, 6, 12 and 24 hpi. **C**, *srsf5*$^{-/-}$ HEK293 cells were transfected with SRSF5-Flag plasmids or empty control for 24 h and infected with PR8 virus at 1.0 MOI. Splicing ratios of M and NS mRNAs were derived from RT-qPCR at the indicated time points. Data presented as means ± SD. **D**, Relative ratio of M2 mRNA in cytoplasm and nucleus from the infected *srsf5*$^{+/+}$ and *srsf5*$^{-/-}$ HEK293 cells at 6 and 12 hpi. Data presented as means ± SD. Data **B**, **C** are representative of three independent experiments. Statistical significance in **C** was determined by unpaired two-tailed Student's t-test.

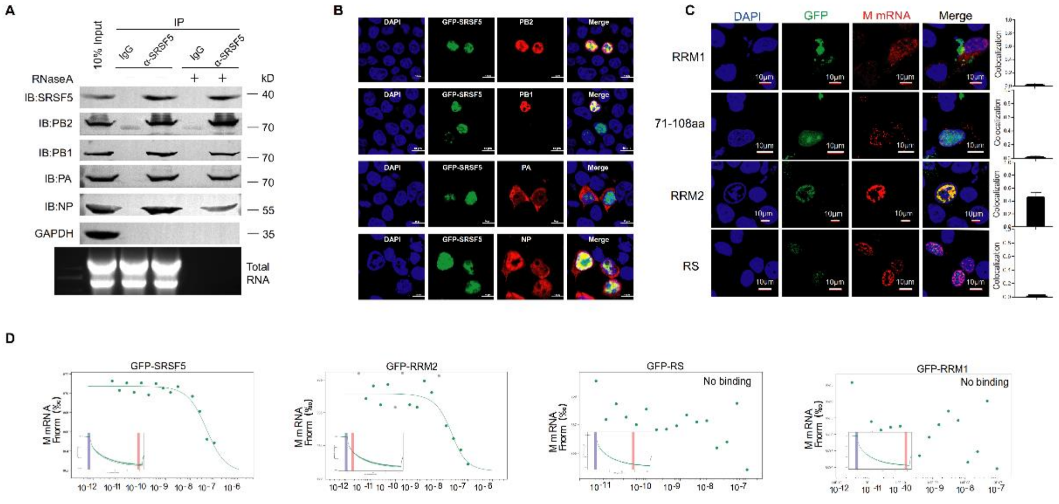

Figure S3. SRSF5 interacted with influenza vRNP and directly bound M pre-mRNA via its RRM2 domain. A, A549 cells infected with PR8 virus at 1.0 MOI for 12h, followed by co-immunoprecipitation (co-IP) with anti-SRSF5 or IgG and immunoblotting with PB2, PB1, PA and NP antibodies in the presence or absence of RNase A. **B**, HEK293 cells were transfected with GFP-tagged SRSF5 plasmid along with RFP-tagged PB2, RFP-tagged PB1, RFP-tagged PA or RFP-tagged NP plasmid for 24 h. SRSF5 (green) clearly co-localized with PB2, PB1 and NP but not with PA.

Scale bars, 10 µm. **C**, A549 cells were transfected with GFP-tagged SRSF5 (full length) or GFP-tagged mutated SRSF5 plasmids (as indicated) for 24 h and infected with PR8 virus at 1.0 MOI for 12 h. Co-localization of full-length or mutated SRSF5 (green) with viral M mRNA (red, detected by RNA FISH). Nuclei were stained with DAPI (blue). Scale bars, 10 µm. **D**, HEK293 cells were transfected with GFP-tagged SRSF5 (full length) or GFP-tagged mutated SRSF5 plasmids (as indicated) for 24 h. Cell lysates were incubated with influenza M mRNA. Binding affinity as determined by MST assays indicated that M mRNA was able to bind full length SRSF5 and its RRM2 domain. For **A-D** data are representative of three independent experiments.

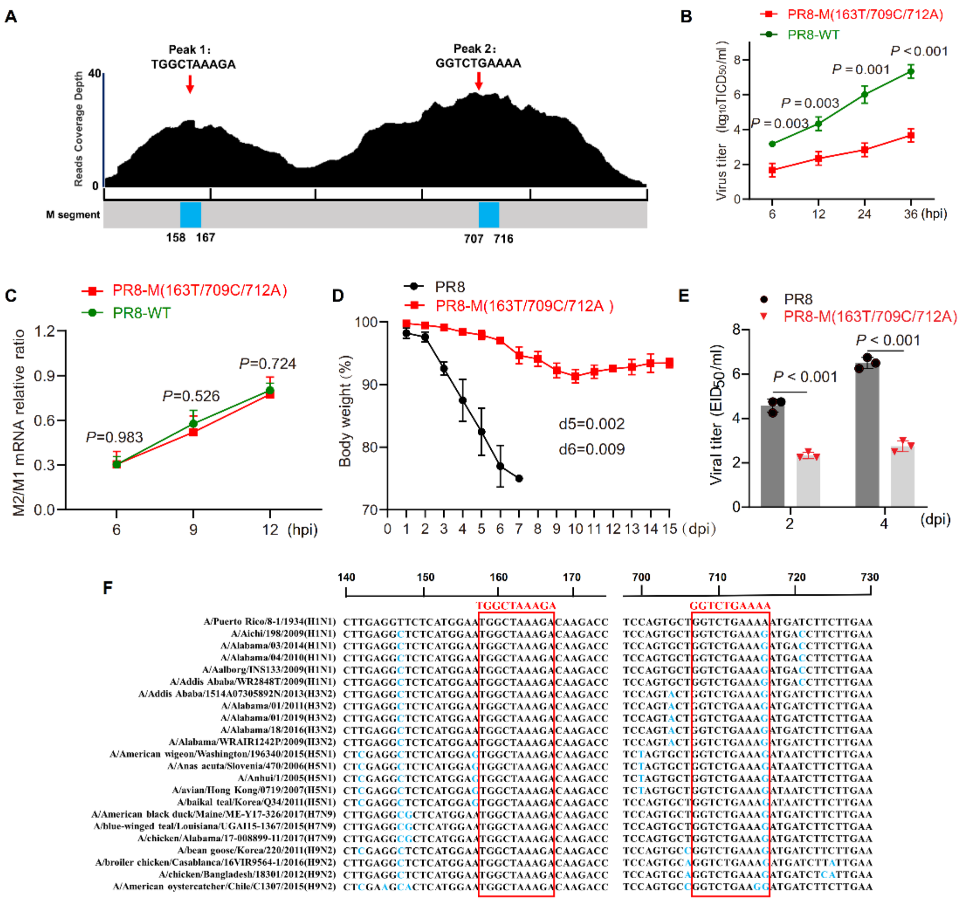

Figure S4. Identification and analysis of binding sites in RRM2 domain of M mRNA. A, Integrated Genome Viewer of captured M segments peaks from Flag-immunoprecipitations of PR8-infected *srsf5*$^{-/-}$ cells over-expressing RRM2-Flag. **B**,

Viral titers of A549 cells infected with PR8 wild type virus or PR8 mutant (163T/709C/712A) (1.0 MOI) at indicated time points post-infection as determined by $TCID_{50}$ assays. **C**, The M2/M1 mRNA ratios of PR8 wild type and PR8 mutant virus-infected srsf5$^{-/-}$ HEK293 cells at the indicated time points. Data presented as means ± SD. **D**, C57BL/6 mice (n = 8) were infected with 10^3 $TCID_{50}$ of PR8 virus or PR8 mutant virus. Changes in body weight were monitored daily. Data are presented as the mean ± SD. **E**, Viral titres in lung tissues of PR8 or mutant virus-infected mice at 2 and 4 dpi were determined by $TCID_{50}$ assays. Data are from three independent experiments with n = 3 mice per group run in triplicate. Error bars indicate SD. **F**, Sequence alignment of the binding motifs of M gene from H1N1, H3N2, avian H5N1, avian H7N9 and avian H9N2 influenza viruses.

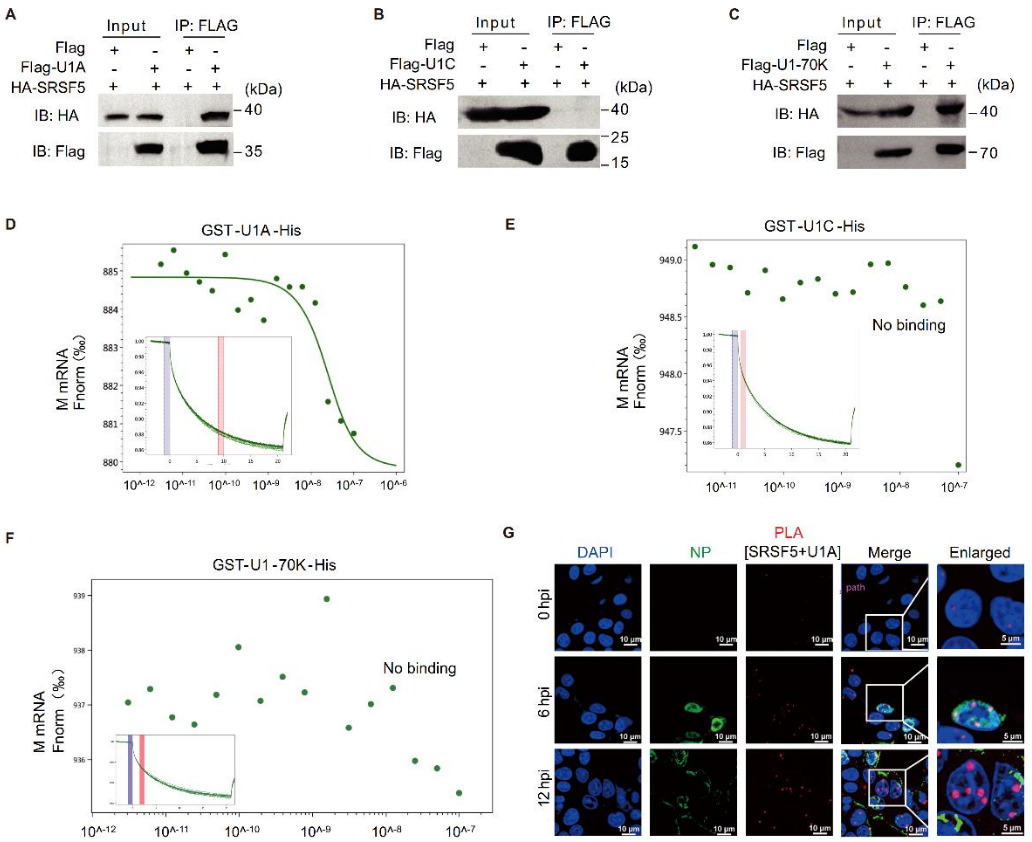

Figure S5. SRSF5 directly interacts with U1A. A, **B**, **C**, HEK293 cells were transfected with Flag-tagged U1A **(A)**, or Flag-tagged U1C **(B)** or Flag-tagged U1-70K **(C),** and HA-tagged SRSF5 plasmids for 24 h, followed by co-IP with anti-Flag

antibody. **D**, **E**, **F**, Purified GST-U1A-his **(D)**, GST-U1C-his **(E)** or GST-U1-70K-his **(F)** was incubated with fluorescein labelled M mRNA respectively. Binding affinity as determined by MST assays. **G**, A549 cells were infected with 1.0 MOI of PR8 virus and subjected to PLA with anti-SRSF5 and anti-U1A antibodies at 0, 6 and 12 hpi. The right column of panels enlarged. Red spots indicate SRSF5 plus U1A complexes, green indicates viral NP protein. For **A-G** data are representative of three independent experiments.

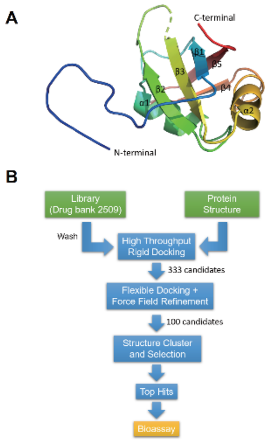

C

Name	Score
Telavancin	13.0809
Tannic acid	12.2771
Colistimethate	12.0728
Fondaparinux	11.6035
Elbasvir	11.2781
Icatibant	11.0661
Anidulafungin	11.0501
Brilliant blue G	10.3784
Ivermectin	9.60564
Rutin	9.25182
Glutathione disulfide	9.15535
Isavuconazonium	9.12146
Tenofovir disoproxil	9.10473
Indocyanine green acid form	8.79092
Bisoctrizole	8.70574
Tazemetostat	8.58752
Novobiocin	8.25629

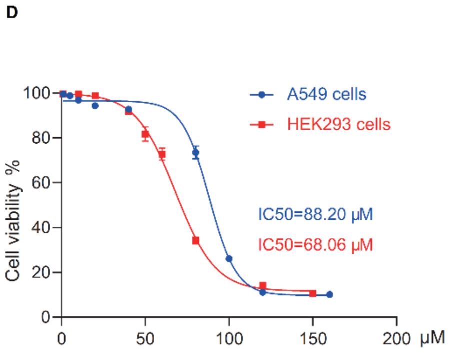

Figure S6. Virtual screening of small molecules against SRSF5 from FDA-approved drugs database. **A**, Predicted model of SRSF5 by homology modeling. **B**, Flowchart of structure-based virtual screening workflow. **C**, High docking score of shortlisted top 17 potential small molecules. **D**, Cell viability of anidulafungin on A549 or HEK293 cells. Data **D** is representative of three independent experiments.
